# Dynamic Detection of Monocyte Subsets in Peripheral Blood of Patients with Acute Hypertriglyceridemic Pancreatitis

**DOI:** 10.1155/2019/5705782

**Published:** 2019-06-10

**Authors:** Junyuan Zheng, Junjie Fan, Chunlan Huang, Yingying Lu, Zehua Huang, Xingpeng Wang, Yue Zeng

**Affiliations:** ^1^Department of Gastroenterology, Shanghai General Hospital, Shanghai JiaoTong University School of Medicine, Shanghai, China; ^2^Shanghai Key Laboratory of Pancreatic Disease, Shanghai JiaoTong University School of Medicine, Shanghai, China

## Abstract

**Aim:**

Monocytes play an important role in acute pancreatitis (AP). Hypertriglyceridemic pancreatitis (HTGP) is always more severe than normal lipid-AP, whether the mechanism of aggravation involves monocyte subsets remains unknown though. The present study was aimed to analyze changes of peripheral blood M1 and M2 monocytes in HTGP patients.

**Methods:**

A total of 90 subjects were enrolled, among which 16 diagnosed with HTGP, 34 with acute biliary pancreatitis (ABP), 20 with hypertriglyceridemia (HTG), and 20 healthy controls (HC). Peripheral blood CD14+CD86+ M1 and CD14+CD206+ M2 monocytes were examined by flow cytometry on days 1, 3, and 7 after admission.

**Results:**

We found a marked increase in total and M1 monocyte count in AP patients (*P* < 0.05). In HTGP, the percentage of M1 monocytes in white blood cells was significantly higher on days 1, 3, and 7, while M2 monocyte percentage was decreased on day 3, compared with ABP (*P* < 0.05). In mild HTGP, M1 monocyte count and percentage gradually decreased, while M2 monocyte percentage gradually increased from day 1 to 7. In severe HTGP, M1 monocyte count and percentage rose to the highest point while M2 were the lowest on day 3. Additionally, the level of M1 monocytes showed a positive correlation with plasma triglyceride and Ranson score of HTGP patients.

**Conclusions:**

Peripheral blood M1 and M2 monocytes showed different dynamic changes in mild and severe HTGP. A more dominant role of CD14+CD86+ M1 monocytes may be involved in the pathogenesis of HTGP.

## 1. Introduction

Hypertriglyceridemia (HTG) is the third most common etiology of acute pancreatitis (AP), secondary to gallstones and alcohol abuse [1]. AP patients are generally categorized as having hypertriglyceridemic pancreatitis (HTGP) when triglyceride (TG) level exceeds 1000 mg/mL or between 500 and 1000 mg/mL but with lipemic serum [2]. Both clinical and experimental studies have demonstrated that compared with acute biliary pancreatitis (ABP), HTGP is more severe and displays aggravated inflammation [3, 4]. However, the exact mechanism remains unknown.

In recent years, much emphasis has been put on the role of immunocompetent cells, particularly monocytes and macrophages, in the progression of AP [5, 6]. Monocytes are an important type of white blood cells (WBCs) and differentiate into tissue macrophages when they leave the circulation system. Human peripheral blood monocytes can be activated as M1 or M2 subsets. M1 monocytes are considered proinflammatory and characterized by expressing proinflammatory cytokines such as interleukin (IL)-6, IL-1*β* and tumor necrosis factor-*α* (TNF*α*), while M2 monocytes produce anti-inflammatory cytokines (e.g., IL-10) and other regulatory cytokines such as transforming growth factor-*β* (TGF*β*). M1 and M2 subsets express different activation markers, among which CD68 and CD206 are routinely used to identify the M1 and M2 phenotype, respectively [7, 8]. Different changes of polarized monocytes are associated with diseases. Zhang et al. [9] reported increased numbers of CD14+CD163- monocytes, CD14+CD163-MAC387+ M1 monocytes, and CD14+CD163+CD115+ M2 monocytes in patients with new-onset mild AP, the latter was also suggested to be important factor in determining the severity and prognosis of severe AP [10]. Bonjoch et al. [11] have demonstrated that in acute pancreatitis rats, lipids from visceral adipose tissue interfered on the M2 polarization of macrophages and promoted the switch to a more intense proinflammatory M1 response, suggesting an association between lipids and macrophage polarization. However, so far, little is known about the changes of peripheral blood M1 and M2 monocytes in HTGP.

Therefore, our study aimed to analyze the dynamic changes of M1 and M2 monocytes in peripheral blood of patients with HTGP and explore how monocytes participate in the pathogenesis of HTGP.

## 2. Materials and Methods

### 2.1. Patients

Of the patients with AP who were admitted to Shanghai General Hospital in China from January 2017 to May 2017, 50 were included in our study prospectively. Age and sex matching non-AP subjects were chosen as controls, including 20 healthy controls (HC) and 20 with HTG. Only those with at least moderate HTG, namely a TG level of ≥2.3 mmol/L (200 mg/mL) [12], were included in the HTG group.

All patients met the Atlanta criteria of AP [13]. Transabdominal ultrasonography or computed tomography (CT) was performed to diagnose ABP. An alanine aminotransferase (ALT) level of >150 U/L within 48 h after the onset of symptoms was used to confirm ABP [14]. AP patients with a TG level of ≥11.30 mmol/L (1000 mg/mL) or between 5.65 and 11.30 mmol/L (500 and 1000 mg/mL) but with lipemic serum were diagnosed with HTGP [2]. AP was classified into three degrees of severity according to the revised Atlanta classification: mild AP (MAP), moderately severe AP (MSAP), and severe AP (SAP) [13]. However, to more clearly clarify the role of monocytes in AP, only MAP and SAP were included in our study, with MSAP excluded. Patients were excluded if he/she had a history of autoimmune diseases, allergy, malignant tumor, or chronic inflammatory diseases.

The study protocol was approved by the Ethics Committee of Shanghai General Hospital and was performed in accordance with the ethical standards. Informed consent was obtained from all subjects.

### 2.2. Clinical Data

The clinical data of each subject was collected from hospital records. These data included age, sex, and laboratory tests. Each individual was subjected to routine laboratory tests for full blood cell counts, the levels of TG, and total cholesterol (TC), ALT. Ranson score, APACHE II (Acute Physiology and Chronic Health Evaluation) score, and Balthazar score [6] were evaluated.

### 2.3. Flow Cytometric Analysis

Peripheral fresh blood samples anticoagulated with EDTA were collected on the 1st, 3rd, and 7th day after admission. Samples from non-AP subjects were taken only once. Each blood sample was disposed of within 30 min. Erythrocytes were lysed according to the manufacturer's instructions (Red Cell Lyse Buffer, Beyotime Biotechnology, China). Leukocytes were immunophenotyped for CD14, CD68, and CD206. Fluorescein isothiocyanate (FITC)-CD14, phycoerythrin (PE)-CD86, and allophycocyanin (APC)-CD206 monoclonal antibody were all purchased from eBioscience, USA. After incubation with 5 *μ*L of the respective antibody solution for 30 min at 4°C in the dark, the cells were washed twice with PBS. Then, fluorescently labeled monocytes were analyzed on the flow cytometer. CD14 is the typical marker for monocytes. M1 cells were characteristically CD86-positive and M2 cells CD206-positive.

### 2.4. Statistical Analysis

Data are presented as means ± SEM. A one-way analysis of variance (ANOVA), the nonparametric Mann-Whitney *U* test and Spearman's test were performed. All the statistical analyses were performed by the SPSS 19.0 software. *P* < 0.05 was considered statistically significant.

## 3. Results

### 3.1. General Information of Study Subjects

The characteristics of study subjects are shown in Table 1. 34 patients with ABP and 16 with HTGP met the inclusion criteria in the study. The age and gender distribution were statistically consistent among HC, HTG, ABP, and HTGP groups. The overall severity of ABP and HTGP showed no significant difference. We also analyzed the patients with mild ABP (MABP), mild HTGP (MHTGP), severe ABP (SABP), and severe HTGP (SHTGP), respectively, as shown in Table 2.

### 3.2. Increased Level of CD14+CD86+ M1 Monocytes in HTGP Patients

**Figure 1:**
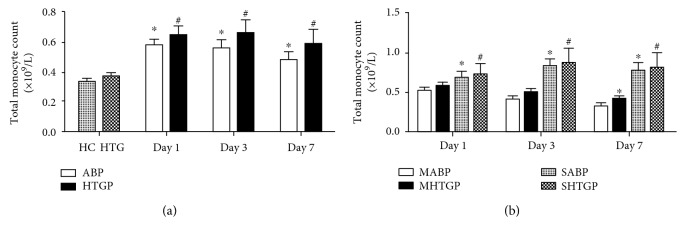
Peripheral blood total monocyte count in all subjects on days 1, 3, and 7. ^∗^*P* < 0.05 compared with HC group (a) or MABP group (b). ^#^*P* < 0.05 compared with HTG group (a) or MHTGP group (b).

As shown in Figure 1(a), total monocyte count significantly increased on days 1, 3, and 7 in ABP and HTGP groups, compared to that in HC and HTG groups, respectively (*P* < 0.05). The HTGP group showed an elevated level of total monocyte count than that of the ABP group but without statistical difference. SAP patients had significantly higher numbers of total monocytes than MAP patients (*P* < 0.05) (Figure 1(b)).

**Figure 2:**
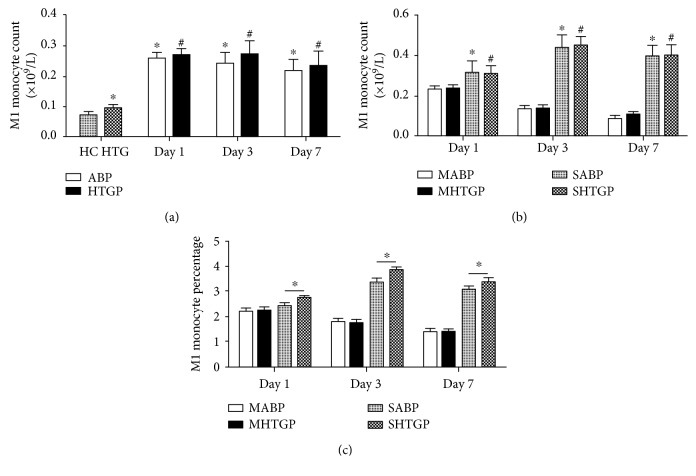
M1 monocyte count and percentage in all subjects on days 1, 3, and 7. ^∗^*P* < 0.05 compared with HC group (a) or MABP group (b). ^#^*P* < 0.05 compared with HTG group (a) or MHTGP group (b). ^∗^*P* < 0.05 (c).

Then, we analyzed CD14+CD86+ M1 monocytes. The HTG group displayed a higher level of M1 monocyte count than the HC group (*P* < 0.05). ABP and HTGP groups showed a significantly increased level of M1 monocyte count on days 1, 3, and 7, compared to the HC and HTG groups, respectively (*P* < 0.05). The HTGP group had an elevated level of M1 monocyte count than that of the ABP group but without statistical difference (Figure 2(a)). M1 monocyte count and percentage were significantly increased in SAP patients than those in MAP (*P* < 0.05) (Figures 2(b) and 2(c)). Most importantly, the percentage of M1 monocytes in white blood cells was significantly increased in the SHTGP group, compared with that in the SABP group on days 1, 3, and 7 (*P* < 0.05) (Figure 2(c)).

Next, we examined a dynamic change trend of M1 monocytes in MAP and SAP, respectively. In MABP and MHTGP groups, M1 monocyte count and percentage were the highest on day 1, then gradually decreased until day 7. In contrast, in SABP and SHTGP groups, M1 monocyte count and percentage reached the peak on day 3 and decreased slightly on day 7.

### 3.3. Decreased Level of CD14+CD206+ M2 Monocytes in HTGP Patients

**Figure 3:**
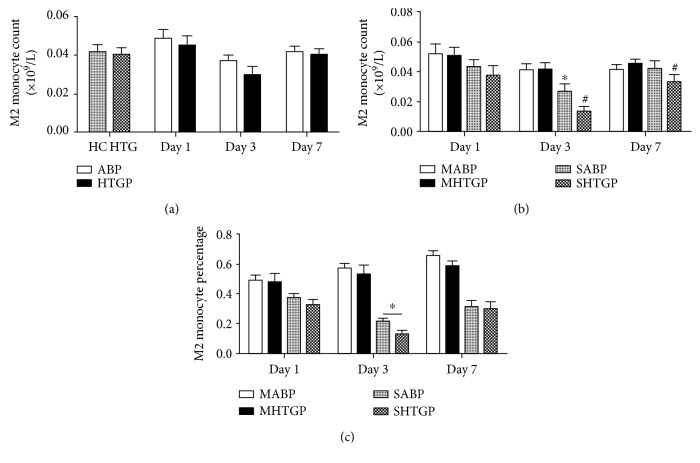
M2 monocyte count and percentage in all subjects on days 1, 3, and 7. ^∗^*P* < 0.05 compared with HC group (a) or MABP group (b). ^#^*P* < 0.05 compared with HTG group (a) or MHTGP group (b). ^∗^*P* < 0.05 (c).

As shown in Figure 3(a), ABP and HTGP groups showed no obvious differences in the CD14+CD206+ M2 monocyte count compared with that of HC and HTG groups, respectively. Severe AP patients had a lower number of M2 monocytes than mild AP patients, with statistical differences on day 3 (*P* < 0.05). Compared with the MHTGP group, M2 monocyte count was significantly decreased in the SHTGP group on day 7 (*P* < 0.05) (Figure 3(b)). Compared with the SABP group, the percentage of M2 monocytes in white blood cells showed a marked decrease in the SHTGP group on days 3 and 7 (*P* < 0.05) (Figure 3(c)).

In MABP and MHTGP groups, M2 monocyte percentage presented a gradual increase trend from day 1 to 7. In SABP and SHTGP groups, M2 monocyte count and percentage decreased to the lowest level on day 3, then recovered on day 7 to the same level as day 1.

### 3.4. M1 Monocyte Level Was Positively Correlated with Plasma TG and Ranson Score in HTGP

**Figure 4:**
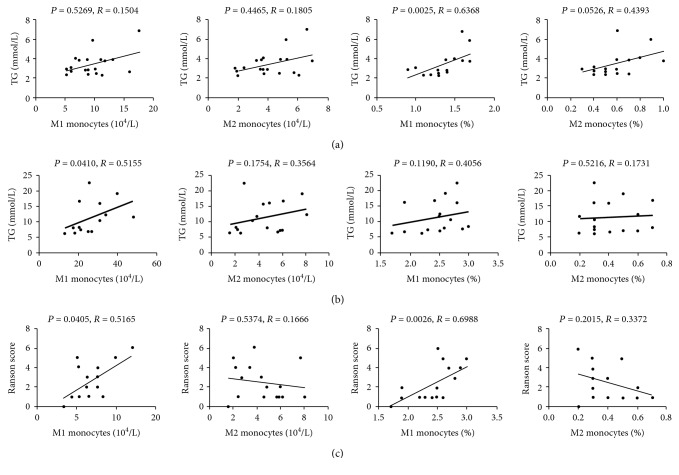
Correlation analysis of monocyte subsets with clinical parameters by spearman test. Association between plasma level of TG and M1 and M2 monocyte count and percentage in the (a) HTG group and (b) HTGP group. (c) Association between Ranson score and M1 and M2 monocyte count and percentage in the HTGP group.

To understand the link between monocyte subsets (day 1) and HTGP pathogenesis, we measured their potential association with plasma level of TG. As seen in Figure 4(a), in the HTG group, M1 monocyte count had no correlation with TG, but M1 monocyte percentage showed a positive correlation with TG level (*P* = 0.0025, *R* = 0.6368), whereas, in the HTGP group, M1 monocyte count was positively associated with TG level (*P* = 0.0410, *R* = 0.5155) (Figure 4(b)). Both M1 monocyte count and percentage were positively correlated with Ranson score (*P* = 0.0405, *R* = 0.5165; *P* = 0.0026, *R* = 0.6988, respectively) (Figure 4(c)). In addition, we did not find any association between M2 monocyte level and plasma TG or Ranson score in the HTGP group (Figures 4(b) and 4(c)). Altogether, our data suggested that CD14+CD86+ M1 subset may be dominantly involved in the pathogenesis of HTGP.

## 4. Discussion

AP is an inflammatory disorder with a complex cascade of immunologic events. The role of immune cells in AP has always been the focus of researchers. Monocytes play an important part in the pathogenesis of AP, and their activation is associated with AP severity [15]. They are generally categorized into two kinds of polarized or functional states. M1 polarization is the classic proinflammatory subtype characterized by the release of proinflammatory mediators contributing to severe inflammation, while M2 monocytes mainly produce anti-inflammatory molecules that control inflammation and promote tissue repair. Transformation of different phenotypes of monocytes regulates the initiation, development, and cessation of various diseases, particularly the inflammatory conditions. Previous reports have reported an increase of M1-polarized monocytes at the early stage of AP [6, 9]. In our study, we consistently observed a marked increase in the numbers of total monocytes and M1 monocytes in AP patients, compared with non-AP controls. Also, for the first time, we found that the percentage of M1 monocytes in white blood cells was significantly higher on days 1, 3, and 7, while M2 monocyte percentage was decreased on day 3 in HTGP, compared with ABP. Furthermore, M1 monocyte level was positively correlated with plasma TG and Ranson score in HTGP patients.

It is reported that lipids are associated with monocyte polarization and activation [16, 17]. HTG may induce a disturbance of monocyte homeostasis [18]. Bonjoch et al. [11] reported that lipids from visceral adipose tissue in AP rats interfere on the M2 polarization of macrophages and promote M1 polarization, resulting in more intense proinflammatory responses. Accordingly, in the present study, we found a statistically higher level of M1 monocyte count and a slightly decreased level of M2 monocyte count in the HTG group, compared with the HC group. M1 monocyte percentage was even positively correlated with TG level in the HTG group. Perhaps this partly explains why HTG, even mild-to-moderate level, is associated with high risk of AP [19]. TG-mediated lipotoxicity is acknowledged to contribute to the aggravation of HTGP. Elevated serum TG in AP patients are independently and proportionally correlated with persistent organ failure regardless of etiology [20]. High TG level in HTGP patients may be associated with adverse prognosis including higher mortality rate [21]. We propose that HTG may aggravate AP via TG-mediated lipotoxicity on monocyte polarization. HTG acts as the first hit and facilitates proinflammatory M1 monocyte polarization, leading to the amplification of proinflammatory responses in HTGP.

On the other hand, through dynamic detection, we observed different dynamic changes of M1 and M2 monocytes in mild and severe HTGP. In MHTGP, the number and percentage of M1 monocytes gradually decreased from day 1 to 7, M2 monocyte percentage gradually increased from day 1 to 7, whereas, in SHTGP, monocyte subsets displayed completely contrary changes. M1 monocyte count and percentage rose to the highest point on day 3 and M2 reached the lowest on day 3.

Increased M1 polarization and simultaneously decreased M2 polarization are reported in various conditions such as sepsis, atherosclerosis, cardiovascular diseases, and kidney injury [22, 23]. Different monocyte subsets always dominate under different conditions or in the different stages of the same disease. For instance, during the early stage of bacterial infection, macrophages in the affected tissues are deliberated to be polarized toward an M1 phenotype, which produce a large amount of proinflammatory mediators causing a cytokine storm, thereby contributing to the aggravation of diseases. In order to counteract the excessive inflammatory response, macrophages polarize to an M2 phenotype to protect from excess injury and facilitate recovery [22]. As a result, M2 subset usually dominants the late stage of diseases. We suppose that the theory also applies to peripheral blood monocyte polarization in HTGP. When proinflammatory M1 level went up, anti-inflammatory M2 level correspondingly went down, indicating severe immune dysregulation.

## 5. Conclusions

In summary, our study suggests profound disturbances of peripheral blood monocyte polarization in HTGP. Peripheral blood total monocyte and M1 level were significantly increased, while M2 level was dramatically decreased in HTGP than in ABP. Mild and severe HTGP had different dynamic changes of monocyte subsets. In mild HTGP, M1 level gradually decreased and M2 level gradually increased from day 1 to 7. In severe HTGP, M1 level reached the highest on day 3 and simultaneously M2 was the lowest. Additionally, M1 monocytes showed a positive correlation with plasma TG and Ranson score of HTGP patients. It seems that a more dominant role of CD14+CD86+ M1 monocytes may be involved in the pathogenesis of HTGP. To the best of our knowledge, this was the first study on the numbers and percentage of peripheral blood M1 and M2 monocytes in patients with HTGP. Our findings may provide new insights into the pathogenesis and immunoregulation of HTGP. We also recognized that our study had limitations, such as lack of functional analysis of monocytes and a relatively small sample size. Further investigations on the values of different monocyte subsets in a bigger population are necessary to understand their roles in the pathogenesis of HTGP.

## Figures and Tables

**Table 1:** General information of studied participants.

	Control	HTG	ABP	HTGP	*P* value
No.	20	20	34	16	
Age (years)	46.85 ± 3.91	52.65 ± 1.87	48.59 ± 3.06	42.25 ± 2.51	>0.05
Sex (male/female)	10/10	10/10	17/17	8/8	>0.05
No. (MAP/SAP)			22/12	9/7	>0.05
TG (mmol/L)	1.07 ± 0.07	3.39 ± 0.26	1.24 ± 0.11	11.42 ± 1.30	<0.05
TC (mmol/L)	4.21 ± 0.19	4.93 ± 0.30	4.32 ± 0.23	7.22 ± 0.82	<0.05
ALT (U/L)	21.45 ± 1.93	37.10 ± 3.28	189.90 ± 6.70	53.88 ± 3.77	<0.05
Ranson			2.12 ± 0.26 (0-6)	2.50 ± 0.46 (0-6)	>0.05
APACHE II			3.82 ± 0.50 (0-13)	3.38 ± 0.55 (1-8)	>0.05
Balthazar					
A			11	5	
B			11	4	
C			7	4	
D			4	2	
E			1	1	

**Table 2:** Clinical characterization of patients with MABP, MHTGP, SABP, and SHTGP.

	MABP	MHTGP	SABP	SHTGP
No.	22	9	12	7
Sex (male/female)	10/12	6/3	7/5	2/5
Age (years)	48.27 ± 3.93	44.78 ± 3.02	49.17 ± 5.03	39.00 ± 4.16
Ranson	1.23 ± 0.15 (0-2)	1.11 ± 0.20 (0-2)	3.75 ± 0.35 (3-6)	4.29 ± 1.11 (3-6)
APACHE II	2.86 ± 0.46 (0-7)	2.11 ± 0.39 (1-4)	5.58 ± 0.98 (1-13)	5.00 ± 0.82 (2-8)
Balthazar				
A	11	5	0	0
B	11	4	0	0
C	0	0	7	4
D	0	0	4	2
E	0	0	1	1

## Data Availability

The data used to support the findings of this study are available from the corresponding author upon request.
